# Contribution to the Arabo-Saharan psammophilous Saprininae, with descriptions of two new species (Coleoptera, Histeridae, Saprininae, Hypocaccini)

**DOI:** 10.3897/zookeys.1249.160249

**Published:** 2025-08-25

**Authors:** Yejun Zhang, Tomáš Lackner

**Affiliations:** 1 School of Life Sciences, Liaoning Normal University, Ganjingzi District, Dalian City, Liaoning Province, China Liaoning Normal University Dalian City China; 2 Department of Environmental Systems Science, ETH Zürich, Weinbergstrasse 56, 8092 Zürich, Switzerland Department of Environmental Systems Science, ETH Zürich Zürich Switzerland

**Keywords:** Arabo-Saharian Region, clown beetles, Coleoptera, Histeridae, Hypocaccini, identification key, new taxa, Saprininae, taxonomy

## Abstract

Two new psammophilous Saprininae, *Neopachylopus pharkidodes***sp. nov.** and *N. penatii***sp. nov.** from the Arabian Peninsula and Algerian Sahara, respectively, are described and figured herein. Both are tentatively assigned to the genus *Neopachylopus* Reichardt, 1926, pending further studies. A key to species is given. The second known exemplar of the very rare *Exaesiopus therondi* Lackner, 2015 from the United Arab Emirates is reported and figured.

## ﻿Introduction

The Arabo-Saharan (or Saharo-Arabian) Region is a vast stretch of semi-arid to arid, mostly barren land in the Holarctic Realm covered by hot deserts, semideserts and savanna. The region occupies temperate parts of the Sahara Desert, Sinai Peninsula, Arabian Peninsula and Lower Mesopotamia (Leonovich 1986). The psammophilous Saprininae (Coleoptera: Histeridae) of this vast realm constitute several distinct genera (with notable exceptions) and have been subjected to several, mostly taxonomic studies (e.g., de Peyerimhoff 1936; Olexa 1990; Lackner 2011, 2012, 2013, 2014, 2015). Lackner et al. (2019) sought to define the different degrees of psammophily (sand-association) in Saprininae, with the so-called “ultra-psammophiles” genera *Philothis*, Reichardt, 1930 *Ctenophilothis* Kryzhanovskij, 1987, *Xenonychus* Wollaston, 1864, and *Xenophilothis* Kryzhanovskij, 1987 being morphologically most adapted to life in deep sand and only rather laboriously collected, involving digging in deep sand. The degree of morphological adaptation to sand in several other genera (the so-called “semi-psammophiles”) is less pronounced. Yet, members of the genera *Reichardtiolus* Kryzhanovskij, 1959 or *Alienocacculus* Kanaar, 2008, are likewise linked to life in sand (living inland, distant from the coast), albeit they can occasionally be collected using pitfall traps. Another group of “semi-psammophiles” occurs on beaches, where they prey upon larvae (and adults?) of tiny arthropods, especially flies, living under coastal wrack. Their legs are often swollen to accommodate a larger number of muscles needed to offset the effort required when digging through wet sand. Here belong members of the genera *Hypocaccus* C. Thomson, 1867, *Exaesiopus* Reichardt, 1926 or *Neopachylopus* Reichardt, 1926. The last-mentioned genus is an arbitrarily constructed entity with members distributed across mainly the Holarctic Realm (Lackner 2010; Lackner and Leschen 2017). Because of its heterogeneity and distribution beyond the Palaearctic, *Neopachylopus* was not treated in Lackner’s review of the Palaearctic Saprininae genera (Lackner 2010). According to the latest phylogenetic study based on morphological as well as molecular characters (Lackner et al. 2024), all above-mentioned taxa belong to the tribe Hypocaccini Lackner, 2024. During the past fifteen years, we have been able to examine a large amount of (Palaearctic) Saprininae housed in various museums and private collections. Here, we describe two of the most striking new semi-psammophile saprinine beetles, which we tentatively include in *Neopachylopus*. New distributional data on a very rare member of *Exaesiopus* are also presented, along with drawings.

## Material and methods

Beetles were relaxed in water overnight and observed and measured under a stereo microscope (Leica MSV-266). Morphological techniques for genitalia extraction follow Lackner (2010). Scanning electron micrographs were taken with a JSM 6301F camera at the laboratory of the Faculty of Agriculture, Hokkaido University, Sapporo, Japan. Digital photographs of male terminalia were taken with a Nikon 4500 Coolpix camera and edited in Adobe Photoshop® CS5. Based on the photographs or direct observations, morphological structures were drawn on a light-box HAKUBA KLV-7000. All illustrations were later scanned and elaborated using Adobe Illustrator® CS5. Colour photographs were taken by Mr František Slamka (Bratislava, Slovakia). Measurements follow Ôhara (1994): APW (anterior pronotal width); PPW (posterior pronotal width); EW (elytral width across humeri); EL (elytral length along suture); PEL (pronotal+elytral length along midline).

Material is deposited in the following collections:

**MNHN**Muséum national d’Histoire naturelle Paris, France (O. Montreuil)

**MNSG** Museo Civico di Storia Naturale Genoa, Italy (M. Tavano)

## Results

### Taxonomy

#### Neopachylopus pharkidodes sp. nov.

Taxon classificationAnimaliaColeopteraHisteridae

B4207EC0-F4CF-50EE-A818-44487E97FE42

https://zoobank.org/00AF758B-A914-48D2-AA7F-A30B4E6BBB07

Figs 1 , 2–7 , 8–12 , 13–19 , 37

##### Material examined.

• ***Holotype***, male, side-mounted on triangular mounting card, right protarsus and left metatarsus broken off, latter glued to the same mounting card as specimen, male genitalia extracted, dismembered and glued to a separate mounting card under the specimen, with the following labels: “QATAR-Madinat Al Shamal / Al Ghariyah 9.III.2003 / 26°04'N, 51°21'E G. Sama” (printed); followed by: “MUSEO GENOVA / ex. coll. G. Sama / (acquisto 2008)” (printed); followed by yellow, pencil-written label “10-205” added by T. Lackner in 2010; followed by red, printed holotype label: “*Neopachylopus* / *pharkidodes* spec. / nov. Det. T. Lackner / et Y. Zhang 2025”. The type is deposited in the Penati collection at MSNG. • ***Paratype*** female, with the following labels: “S. Arabia: / Kamaran I. / 7-11-1903 / Dr. M. Cameron / B.M. 1928-109” (printed-written), followed by printed label “Sea Weed”, followed by a pencil-written label: “*Neopachylopus* / sp.nov. 2009 / Det. T. Lackner”, followed by yellow, pencil-written label: “09-048”, added by T. Lackner in 2009; followed by red, printed paratype label: “*Neopachylopus* / *pharkidodes* spec. / nov. Det. T. Lackner / et Y. Zhang 2025” (MNHN).

##### Description.

**Figure 1.**
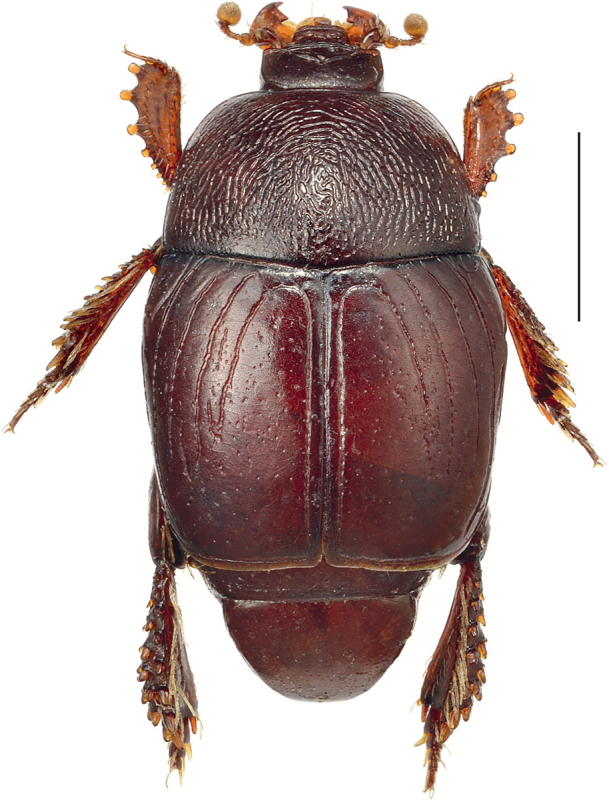
*Neopachylopus pharkidodes* sp. nov., habitus, dorsal view. Scale bar: 1,00 mm.

**Figures 2–7.**
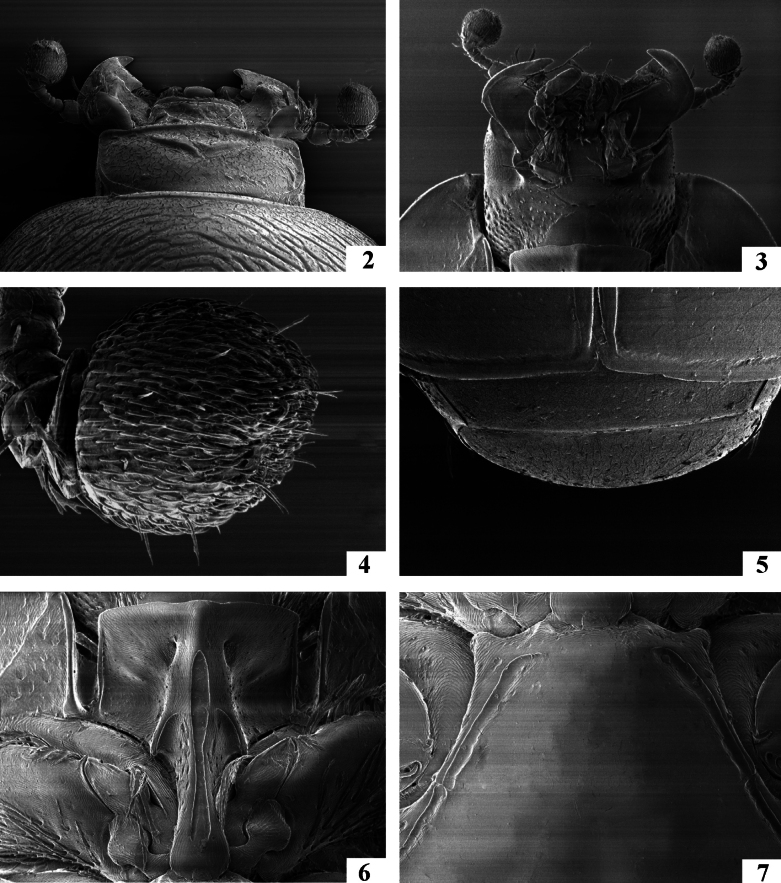
*Neopachylopus pharkidodes* sp. nov. 2. Head, dorsal view; 3. Head, ventral view; 4. Antennal club, dorsal view; 5. Propygidium+pygidium; 6. Prosternum; 7. Mesoventrite.

**Figures 8–12.**
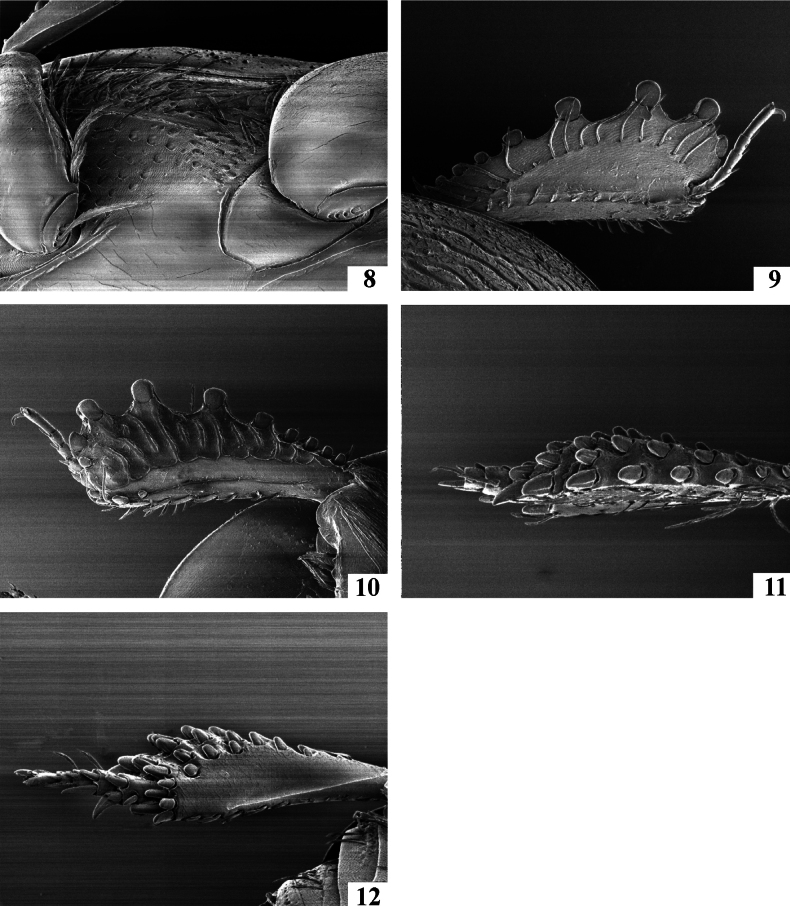
*Neopachylopus pharkidodes* sp. nov. 8. Lateral disk of metaventrite+metepisternum; 9. *Neopachylopus pharkidodes* sp. nov., protibia, dorsal view; 10. *Neopachylopus pharkidodes* sp. nov., ventral view; 11. *Neopachylopus pharkidodes* sp. nov., metafemur, dorsal view; 12. *Neopachylopus pharkidodes* sp. nov., ventral view.

**Figures 13–19.**
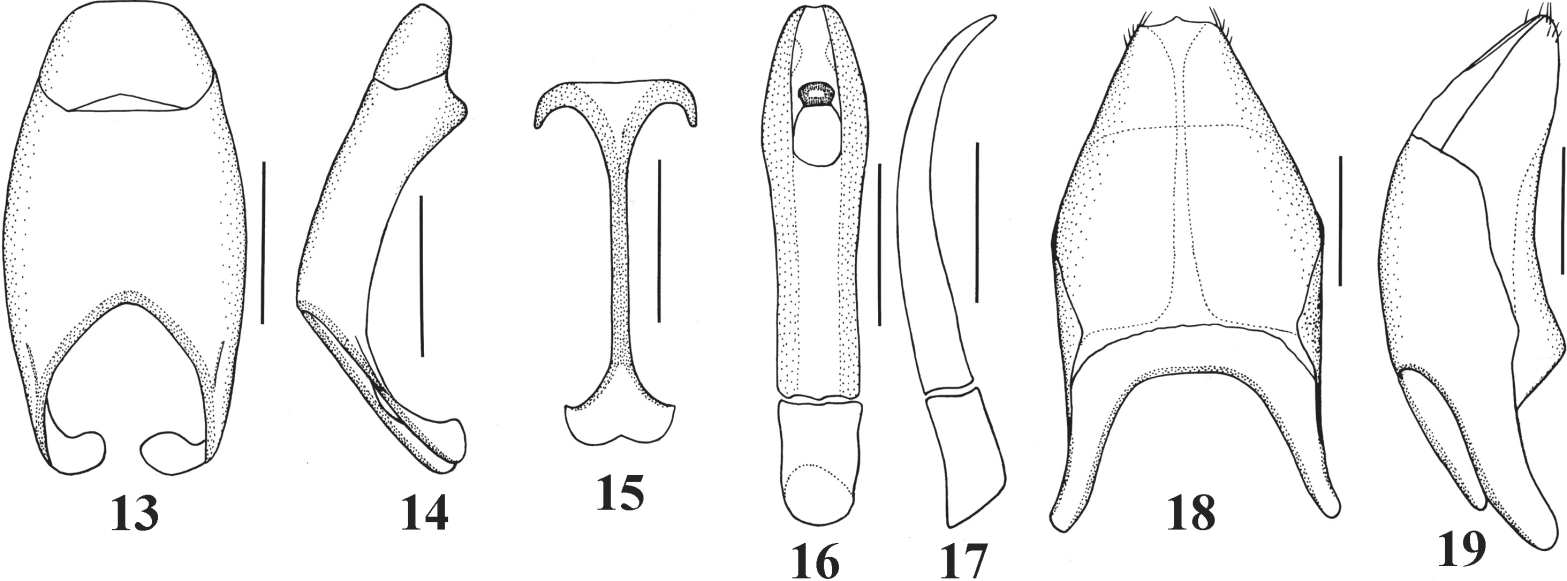
*Neopachylopus pharkidodes* sp. nov. 13. Ninth+tenth tergite, dorsal view; 14. Ninth+tenth tergite, lateral view; 15. Tenth sternite (spiculum gastrale), ventral view; 16. Aedeagus, dorsal view; 17. Lateral view; 18. Eighth sternite+tergite, ventral view; 19. Eighth sternite+tergite, lateral view. Scale bars: 0,1 mm.

Body (Fig. 1): APW: 0.73–0.77 mm; PPW: 1.65–1.69 mm; EW: 1.85–1.91 mm; EL: 1.44–1.48 mm; PEL: 2.35–2.59 mm. Body oval, convex, reddish-brown, dorsal surface of head and pronotum fuscous; body appendages lighter. Head (Fig. 2): frons with microsculpture and indistinct microscopic punctures; medial area with several coarse and intersecting grooves. Frontal stria carinate and complete, anterior part feebly arcuate forward, supraorbital stria extending past posterior margin of eye. Epistoma coarse, carinately margined, depressed medially. Labrum transverse, anterior margin straight medially; setae inconspicuous (broken off?). Mandibles stout, curved, tips acute, left mandible with a distinct sub-apical tooth on inner margin, right mandible with an indistinct sub-apical tooth on inner margin. Mentum (Fig. 3) rectangular, anterior margin inwardly arcuate; eyes flattened, invisible from above. Antennal club (Fig. 4) globose and tomentose, sensory structures not examined. Pronotum (Fig. 1): sides nearly straight and slightly convergent forward in posterior two-thirds, strongly arcuate in anterior third; anterior margin emarginate and evenly arcuate; posterior margin nearly straight laterally, forming an indistinct angle medially. Marginal pronotal stria carinate, complete. Surface densely and coarsely impressed with longitudinal striae and rugae; occasionally intersecting and arcuate medially; a smooth, narrow band along margins present; ante-scutellar area with a single coarse and deep round puncture. Pronotal hypomeron glabrous. Scutellum small, triangular. Elytra (Fig. 1): relatively straight, humeri slightly projecting. Dorsal elytral stria I almost complete, slightly weakened and briefly interrupted on basal third in holotype; dorsal elytral stria II present in basal 2/3; dorsal elytral striae III–IV sub-equal in length, stopping short of elytral half apically; stria IV basally connected with complete sutural elytral stria, which is linked with complete, albeit somewhat weakened apical elytral stria, which, on the other hand is linked to complete and carinate marginal elytral stria. Humeral elytral stria smooth, deeply impressed in basal fourth; inner subhumeral stria present as short median fragment; outer subhumeral stria absent; all striae (except humeral one) feebly crenate. Elytral disk micro-sculptured and irregularly scattered with small punctures. Elytral epipleuron microscopically punctate, almost smooth; marginal epipleural stria deeply and completely impressed. Abdomen: propygidium and pygidium (Fig. 5) with scattered tiny shallow punctures. Prosternum (Fig. 6): anterior margin feebly protruding and slightly extending ventrally, posterior margin nearly straight. Prosternal process narrow, knife-like, carinal prosternal striae very approximate, running sub-parallel, anterior ends joined. Prosternal process with microsculpture and scattered small punctures near anterior margin and laterad of carinal striae; prosternal foveae small, well visible. Lateral prosternal striae short, carinate, convergent and attaining carinal prosternal striae at their apical two-thirds. Mesoventrite (Fig. 7): subtrapezoid; disk glabrous. Marginal mesoventral stria complete and straight laterally, absent anteriorly. Meso-metaventral suture well impressed, medially angulate. Intercoxal disk of metaventrite smooth, like mesoventrite; longitudinal suture deeply impressed, surface around it slightly depressed. Lateral metaventral stria rather short; lateral disk of metaventrite (Fig. 8) slightly depressed, with shallow setigerous punctures. Metepisternum (Fig. 8) with coarser and denser punctures bearing long amber setae. Intercoxal disk of first visible abdominal sternite slightly longer than metaventrite, smooth, incompletely striate laterally. Legs: profemur slightly slender; protibia (Figs 9, 10) broad and flat, outer margin with four teeth bearing blunt denticle, followed by another four tiny denticles entombed in outer protibial margin; all diminishing in size in proximal direction. Anterior face of protibia (Fig. 10) with rib-like structures. Mesofemora stout; mesotibia rather slender, outer margin with two rows of thickly set denticles; mesotarsus relatively thick, slightly longer than protarsus. Metafemora rather stout, sub-semicircular; metatibia (Figs 11, 12) dilated and thickened, outer margin with four rows of densely set short denticles; metatarsus like mesotarsus. Male genitalia: aedeagus (Figs 16, 17) basal piece short, ratio to parameres approximately 1:4; parameres on their basal half (approximately) parallel-sided, thence slightly divergent and convergent again apically, apex blunt; aedeagus slightly curved from ventral view. Rest of male terminalia (Figs 13–15, 18, 19) typical for Hypocaccini; eighth sternite and tergite not fused laterally (Fig. 19); apex of eighth sternite with few short setae.

Female genitalia not studied.

##### Distribution.

Known from Qatar and Yemeni Island of Kamaran; these two localities are geographically widely separated (about 1430 km; Fig. 37); the lack of specimens from between these two points can most likely be attributed to inadequate sampling, thus the species is probably present along the entire coast of the southern Arabian Peninsula.

##### Etymology.

From the Greek word *pharkis*, meaning wrinkle, *pharkidodes*, wrinkled; named after the rugulose pronotal disk.

##### Etymology.

Collected under seaweed.

##### Remarks.

A morphologically unique taxon. The rugulose pronotal dorsum of *N. pharkidodes* sp. nov. is unmatched among all Saprininae taxa known to the senior author (see also Key to species and Discussion).

#### Neopachylopus penatii sp. nov.

Taxon classificationAnimaliaColeopteraHisteridae

8A5C3593-CF7C-56D6-A566-D4A0C014389A

https://zoobank.org/D6972BC0-BFA4-47AD-933B-ADE258D331D3

Figs 20 , 21 , 37

##### Material examined.

• ***Holotype***, female, side-mounted on triangular mounting card, ultimate right protarsomere and left metatarsomere broken off, with the following labels: “female sign” (printed); followed by: “ALGERIE / Sidi Bel Abbés / collection le moult” (printed); followed by: “**Muséum Paris** / **1933** / Coll. Desbordes” (light-turquoise label printed); followed by: “Muséum national / Histoire nat. Paris / coll. générale” (blue label, printed, black-framed); followed by: “*Neopachylopus* / sp. nov. / det. Fabio Penati, 2022” (printed; black-framed label); followed by red, printed holotype label: “*Neopachylopus* / *penatii* spec. nov. / Det. T. Lackner et Y. / Zhang 2025” (MNHN).

##### Description.

**Figures 20, 21.**
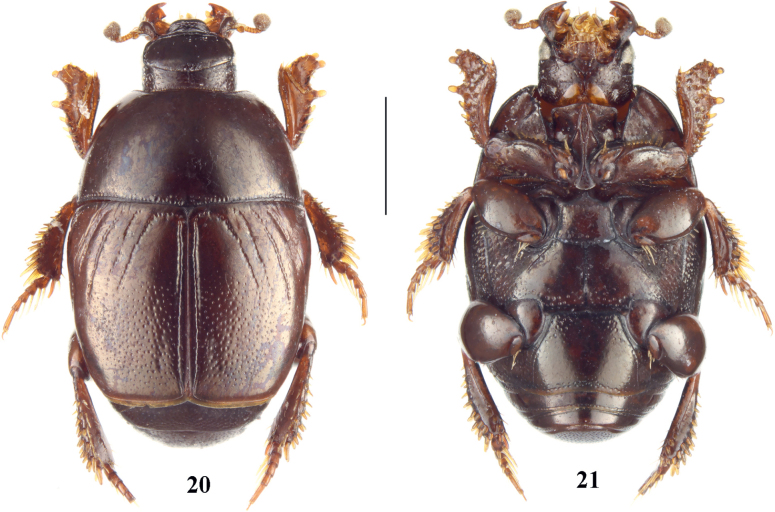
*Neopachylopus penatii* sp. nov. 20. Habitus, dorsal view; 21. Habitus, ventral view. Scale bar: 1,00 mm.

Body (Figs 20, 21) APW: 0.94 mm; PPW: 1.98 mm; EW: 2.11 mm; EL: 1.59 mm; PEL: 2.58 mm: oval, convex, pronotum dark brown, elytra lighter, castaneous; body appendages light brown, antennal club somewhat darker. Head: frontal disk slightly convex, glabrous; frontal stria almost straight, carinate, supraorbital and occipital striae fine, complete. Eyes flattened, almost invisible from above. Clypeus flattened, with several irregular, faint rugae; labrum typical for Hypocaccini, with two erect setae arising from each labral pit. Mandibles light brown, smooth, pointed apically, sub-apical tooth on left mandible large, perpendicular. Rest of visible mouthparts typical for the subfamily. Antennal club rather small, globular, without visible sensory structures; antennal scape with several long, thick yellow setae. Pronotum: pronotal hypomeron glabrous; marginal pronotal stria complete, carinate laterally, slightly weakened behind head. Pronotal disk weakly convergent anteriorly, apical angles blunt; disk glabrous, with several irregular punctures in ante-scutellar area. Elytra: elytral epipleuron glabrous, marginal epipleural stria fine, complete; marginal elytral stria fine, complete, continuous with complete apical elytral stria that is continuous with complete sutural elytral stria. Elytral humeri inconspicuous, humeral elytral stria very thin, present on basal elytral fifth; outer subhumeral stria present as a short median fragment. Dorsal elytral striae I–IV in punctures, of sub-equal length, reaching approximately to elytral third to half apically; stria IV basally connected with sutural elytral stria. Elytral disk on apical half (approximately) with dense, large punctures separated by 0.5–1.5 times their diameter; elytral intervals and flanks impunctate; punctures stopping short of elytral apex, leaving a narrow impunctate band. Propygidium: partly covered by elytra, with dense, deep punctures separated by less than their diameter; interspaces with microsculpture; pygidium triangular, medially convex, with sparser and finer punctures than those of propygidium. Prosternum: very compressed, knife-like, carinal prosternal striae between procoxae very approximate, thence running parallel, diverging and again convergent apically. Lateral prosternal striae short, carinate, attaining carinal prosternal striae at their mid-length; prosternal foveae tiny, rather deep; prosternal process concave. Mesoventrite: subquadrate, mesoventral disk with irregular punctures of unequal sizes; marginal mesoventral stria complete. Meso-metaventral suture with distinct punctate stria. Metaventrite: broad, short, slightly concave medially, intercoxal disk glabrous, near apical margin with a band of irregular punctures of various sizes. Lateral disk of metaventrite with large ocelloid shallow setigerous punctures, interspaces with alutaceous microsculpture. Lateral metaventral stria short, carinate, straight. Metepisternum with very dense, large setigerous punctures. First visible abdominal ventrite completely striate laterally; with irregular punctures of various sizes on basal fourth; rest of disk almost glabrous; along apical edge a row of tiny punctures appears. Legs: protibial apex with two prominent, rounded denticles, followed by a distant short tooth topped by similar denticle, followed by a dense row of six regular, elongate denticles diminishing in size in proximal direction. Protarsal spur short, inconspicuous, anterior protibial margin with three short, thin denticles; protibial groove deep, straight; protarsus thin, ultimate protarsomere shorter than two preceding together; claws short, thin, bent. Mesotibia thickened, outer margin with several rows of dense, thick set denticles on outer margin, growing in distal direction; mesotibial spur long, stout; mesotarsus thickened, each mesotarsomere with long, thick seta; mesotarsal claws thin, bent, shorter than half of ultimate mesotarsomere. Metatibia triangularly dilated and thickened, otherwise like mesotibia; metafemora thickened. Male unknown.

##### Distribution.

Known only from Sidi Bel Abbès, near Oran, north-western Algeria (Fig. 37). Possibly distributed also into Morocco.

##### Etymology.

Named after our colleague Fabio Penati (Morbegno, Italy), who first recognised this unique taxon.

##### Etymology.

Unknown.

##### Remarks.

The glabrous frontal disk, knife-like prosternal process and peculiar shape of protibia (protibial apex with two prominent rounded denticles, followed by a distant short tooth topped by similar denticle) clearly separate this taxon from members of genus *Hypocaccus*, which it superficially resembles most. The smooth, impunctate pronotum would suggest affinity to the subgenus Baeckmanniolus Reichardt, 1926 of the aforementioned genus. Yet by the above-mentioned characters, *N. penatii* sp. nov. cannot be classified within it. Hesitant to erect a new genus, we decided to include it in the heterogeneous *Neopachylopus*, pending further studies (see also Discussion).

### Key to the Palaearctic *Neopachylopus* Reichardt, 1926

**Table:** 

1	Prosternal keel somewhat rounded, not knife-like. Protibia bidentate, pronotal hypomeron fimbriate (see Lackner, 2001: figs 2, 3)	***N. pakistanicus* Lackner, 2001 (southern Pakistan)**
–	Prosternal keel knife-like (Fig. 6); protibia with multiple teeth diminishing in proximal direction (Fig. 9); pronotal hypomeron glabrous	**3**
3	Pronotal disk rugulose (Fig. 1)	***N. pharkidodes* sp. nov. (Qatar, Yemen)**
–	Pronotal disk glabrous (Fig. 22)	**5**
5	Dorsal elytral stria IV and sutural elytral stria not connected basally (Fig. 22)	***N. secqi* Kanaar, 1998 (Djibouti, Yemen, Oman)**
–	Dorsal elytral stria IV basally connected with sutural elytral stria (Fig. 23)	**7**
7	Elytral surface almost smooth; reddish species (Fig. 23)	***N. kochi* Thérond, 1963 (Somalia)**
–	Elytral surface punctate; brownish species (Fig. 20)	***N. penatii* sp. nov. (Algeria)**

### New distributional data

#### Exaesiopus therondi

Taxon classificationAnimaliaColeopteraHisteridae

Lackner, 2015

3B7BDFCF-2801-5956-90A2-A3719F6D4950

Figs 24–36 , 37

##### Material examined.

♂, United Arab Emirates, FUJAIRAH [=Sharjah], Wadi al Helo, SW1, KHOR KALBA, 24°53'833"N, 56°19'875"E, 25.ii.2007, G. Sama lgt., deposited in Penati collection at MSNG. A recently described species, the holotype of which was collected from the stomach of a Kentish plover [*Anarhynchus alexandrinus* (Linnaeus, 1758), Aves: Charadriidae] in Afghanistan (Lackner 2015). This is the second known specimen (also male); new to United Arab Emirates. Its occurrence in the UAE suggests the species is widely spread, most likely present in Iran and Pakistan as well. For its better recognition, we show the morphological characteristics of the Arabian specimen (Figs 24–36).

**Figures 22, 23.**
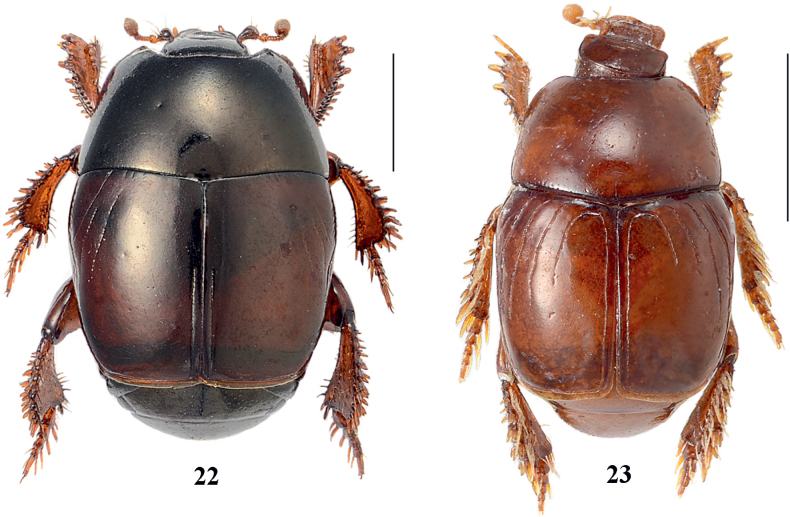
22. *Neopachylopus secqi* Kanaar, 1998, habitus, dorsal view; 23. *Neopachylopus kochi* Thérond, 1963, habitus, dorsal view. Scale bars: 1,00 mm.

**Figures 24–36.**
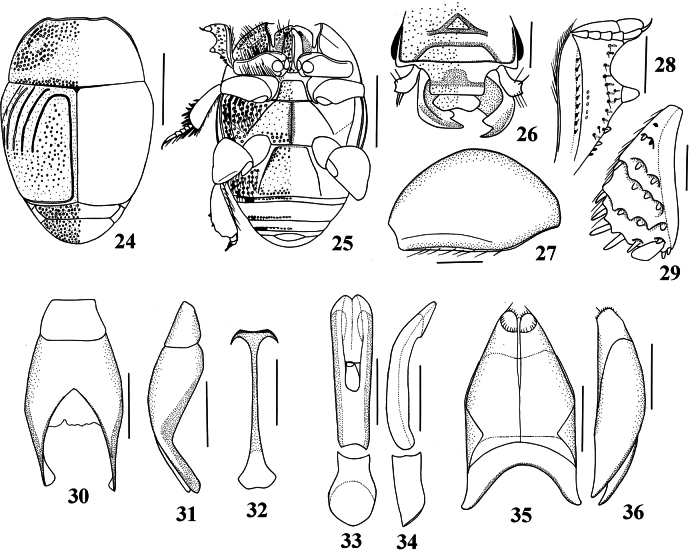
*Exaesiopus therondi* Lackner, 2015. 24. Habitus, dorsal view; 25. Habitus, ventral view; 26. Head, frontal view; 27. Metafemur; 28. Protibia, dorsal view; 29. Metatibia, lateral view; 30. Ninth tergite+sternite, dorsal view; 31. Ninth tergite+sternite, lateral view; 32. Tenth sternite (spiculum gastrale), ventral view; 33. Aedeagus, dorsal view; 34. Aedeagus, lateral view; 35. Eighth sternite+tergite, ventral view; 36. Eighth sternite+tergite, lateral view. Scale bars: 1,00 mm (24, 25); 0,5 mm (26–29); 0,1 mm (30–36).

**Figure 37.**
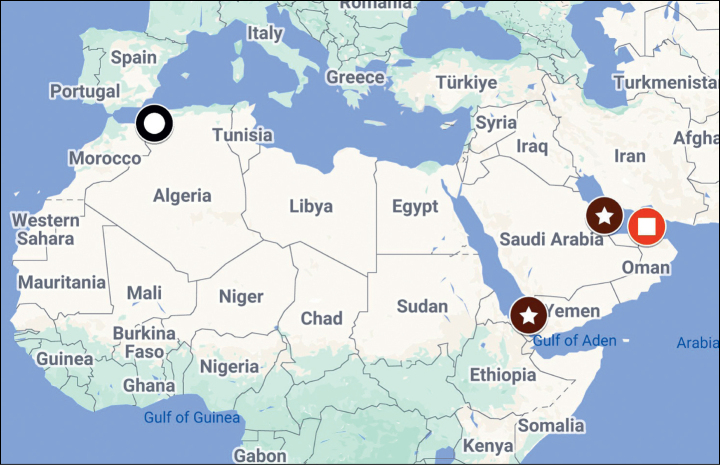
Known distribution of: *Neopachylopus penatii* sp. nov. (empty circle); *Neopachylopus pharkidodes* sp. nov. (circle with a star); *Exaesiopus therondi* Lackner, 2015 (circle with rectangle).

## Discussion

The genus *Neopachylopus* currently contains five described species: two occur on the Pacific Coast of Mexico, USA and Canada (*N. aeneipunctatus* (Horn, 1871) and *N. sulcifrons* (Mannerheim, 1843)); *N. secqi* Kanaar, 1998 is known from the coastal regions of Horn of Africa as well as the southern Arabian Peninsula (Djibouti, Yemen, Oman); *N. kochi* Thérond, 1963 is a Somali endemic and *N. pakistanicus* Lackner, 2001 is so far a Pakistani endemic. According to Kanaar’s (1998) definition, slightly modified here, *Neopachylopus* combines the following characters: a) impunctate pronotum; b) knife-like carinal prosternal striae; c) presence of lateral prosternal striae; and d) strongly thickened and dilated meso-and-metatibiae, their outer margin with several rows of dense denticles. Lackner (2001), however, noted that the members of the genus were morphologically quite dissimilar and that *N. pakistanicus* did not quite fit the generic definition due to the somewhat rounded, not knife-like prosternal process, fimbriate pronotal hypomeron (glabrous in other species) and peculiarly shaped protibia (possessing only two large distal teeth, as opposed to normally shaped protibia of other congeners). Lackner (2001) also noted a lack of phylogenetic resolution in studies involving members of *Neopachylopus* and added, “assignment of *N. pakistanicus* to *Neopachylopus* is questionable”. Lackner et al. (2024) published a phylogeny of the subfamily including three members of the genus: two Nearctic ones formed a clade (100% support), sister to the Arabian *N. secqi*, which formed a strongly supported clade with South African (and Namibian) *Pachylopus dispar* Erichson, 1834; sistership of these two monophyla was only moderately supported. Due to the low resolution of inter-relationships within the Hypocaccini tribe, as well as incomplete sampling, Lackner et al. (2024) did not implement further taxonomic changes in the genus and decided to keep the *status quo*. The two newly described species in this paper stretch the *Neopachylopus* definition further. While *N. pharkidodes* sp. nov. fits the definition of the genus almost completely, it differs from it by the rugulose pronotum, which is unique for the subfamily. *Neopachylopus penatii* sp. nov., on the other hand, does not possess thickened metafemora and metatibiae. We stress that the inclusion of both newly described species in *Neopachylopus* is only tentative pending further, ideally molecular studies of these rare taxa.

## Supplementary Material

XML Treatment for Neopachylopus pharkidodes

XML Treatment for Neopachylopus penatii

XML Treatment for Exaesiopus therondi

## Additional information

### Use of AI

No use of AI was reported.

### Author ORCIDs

Tomáš Lackner https://orcid.org/0000-0002-0108-5785

### Data availability

All of the data that support the findings of this study are available in the main text.
